# Sensory characteristics of vegetables consumed by Australian children

**DOI:** 10.1017/S1368980021000847

**Published:** 2022-05

**Authors:** David N Cox, Danielle L Baird, Megan A Rebuli, Gilly A Hendrie, Astrid AM Poelman

**Affiliations:** 1 CSIRO Health and Biosecurity, Adelaide, SA 5000, Australia; 2 CSIRO Food and Agriculture, North Ryde, NSW, Australia

**Keywords:** Child, Adolescent, Vegetables, Sensory, Preference, Taste perception, Texture perception

## Abstract

**Objectives:**

Consumption is driven by children’s sensory acceptance, but little is known about the sensory characteristics of vegetables that children commonly eat. A greater understanding could help design more effective interventions to help raise intakes, thus realising beneficial health effects. This study sought to: (1) Understand the vegetable consumption patterns in children, with and without potatoes, using the Australian and WHO definitions. (2) Describe the sensory characteristics of vegetables consumed by children by age group, level of intake and variety. (3) Determine the vegetable preferences of children, by age group, level of intake and variety.

**Design:**

Analysis of National Nutrition Survey data, combining reported vegetable intake with sensory characteristics described by a trained panel.

**Setting:**

Australia

**Participants:**

A nationally representative sample of Australian children and adolescents aged 2–17·9 years (*n* 2812).

**Results:**

While consumption increased in older age groups, variety remained constant. Greater variety, however, was associated with higher vegetable consumption. Potato intake increased with consumption, contributing over one-third of total vegetable intake for highest vegetable consumption and for older age groups. Children favoured relatively sweet vegetables and reported lower consumption of bitter vegetables. There were no differences in the sensory properties of vegetables consumed by children in different age groups. After potatoes, carrots, sweetcorn, mixtures, fruiting and cruciferous types were preferred vegetables.

**Conclusion:**

Children tend to prefer vegetables with sensory characteristics consistent with innate taste preferences (sweet and low bitterness). Increasing exposure to a variety of vegetables may help increase the persistently low vegetable consumption patterns of children.

## Introduction

A high intake of vegetables is associated with better health^(1,2)^ and may assist with obesity prevention^(3)^ and yet, worldwide, children do not achieve the recommended amounts. For example, in Australia, there is evidence of sustained low vegetable consumption among children, with only 5 % reporting intakes consistent with recommendations^(4)^. Recent reviews suggest that interventions to increase children’s vegetable consumption in schools^(5)^ and in the broader community^(6)^ have small to modest effects on usual intakes. However, another review^(7)^ that focused on intake of specific target vegetables as an outcome in contrast to usual intakes found positive effects of exposure-type interventions. Australian children’s usual exposure through eating occasions tends to be limited, with dinner (evening meal) the most prevalent occasion for serving vegetables^(8)^. This suggests that more research is needed to understand more about how we can increase vegetable consumption in children.

Drivers of food choice are numerous^(9)^ but there is consensus that taste (as a colloquial term for sensory characteristics) is a primary consideration for most people in nearly all food and drinking settings^(9)^. The innate dislike of bitter taste^(10)^, associated with alkaloid toxins, could be particularly relevant to the acceptance of vegetables given that some, particularly cruciferous types, have salient bitter taste^(11,12)^ associated with their beneficial phytonutrient properties^(13)^. Further, most core food groups have sensory properties which are innately liked or acquired very early in life^(12)^; however, vegetables do not^(12)^ and are relatively low in all taste qualities known to contribute positively to liking, such as sweet and salty tastes and fatty mouthfeel^(12,14)^. Thus, the sensory properties of vegetables are challenging for humans to learn to like^(15)^.

Early work by Baranowski *et al.*^(16)^ found that among fourth and fifth grade students, there was a predominant belief that ‘if a food tastes good, it must not be good for me; and if a food tastes bad, it is probably good for me’. Further work by this group^(17)^ supported this assertion that children thought ‘veggies taste nasty’. Another qualitative study^(18)^ found that food choice amongst UK preschoolers was not determined by the health attributes of food but by preference, play, socialisation and convenience. Another study of school-age children in the UK^(19)^ found that taste was by far the biggest impediment to healthy diets, as healthy food was not positively associated with taste. The children even presumed ‘healthy’ food they had not tried would not be nice. Indeed, a review^(20)^ identified taste preferences and liking as being the most important for European children’s food choices with availability (exposure) driving such preferences and liking through cultural practices and exposure.

Specific studies on health and taste include experimental work by Wardle & Huon^(21)^ demonstrating that simply labelling a beverage as healthy decreased children’s liking due to the association with poor taste. Further testing of the ‘unhealthy equals tasty’ hypothesis used both implicit and explicit measures^(22)^ and found, in a series of experiments, that unconsciously (implicitly) children and adults associated healthy foods (including vegetables) with poor taste. Such a relationship held true amongst participants who explicitly believed that ‘healthy equalled not tasty’ and those that did not believe this. More recent evidence^(23)^ shows that simply giving children ‘instrumental’ information (‘eat this because….’) decreased liking for a range of foods and beverages including vegetables. In summary, evidence suggests that locating vegetable consumption within a health framework is likely to evoke beliefs that such ‘healthy’ foods are unpalatable; hence, using explicit health messages to encourage vegetable consumption might fail because children are driven by the sensory attributes of foods, hence the sensory focus of the current study.

There is an emerging consensus that disliking of sensory characteristics can be overcome through learning^(24,25)^, and that the tastes children are exposed to at an early age have long-lasting effects on their liking of specific tastes^(26)^, consistent with mere exposure effects^(27)^. In addition, children’s taste perceptions differ from adults, with heightened bitter sensitivity and lowered sweetness perception at a young age^(15)^, and age-related changes in perception facilitates learning effects with age. However, it is unknown precisely how this learning, or lack of learning, influences the consumption of an important food category such as vegetables. Given this uncertainty, it is difficult to formulate formal hypotheses, but scenarios can be explored. For example, if liking for vegetables is learned^(24)^, it is possible that younger children eat more vegetables that align with innate likings, that is, sweeter vegetables, but that this changes with age as children learn to like different tastes. Similarly, perhaps children with a lower vegetable intake may eat more vegetables with sensory properties aligned to their innate likes and dislikes (i.e. sweeter and less bitter) and perhaps children with a higher vegetable intake might have learnt to like vegetables with more varied and innately disliked sensory characteristics, as reflected in relatively lower sweet and higher bitter characteristics. The same may apply to the variation in the variety of vegetables eaten. Conversely, children consuming a greater amount of vegetables may have a high intake of more innately palatable vegetable options.

In the current study setting, recommendations within the Australian Dietary Guidelines (ADG) regarding vegetable consumption^(28)^ include starchy roots and tubers. This contrasts with recommendations from the WHO^(29)^ and most other jurisdictions that have adopted the WHO definitions that do not include starchy roots and tubers as ‘vegetables’. The reasons why the ADG include starchy roots and tubers (most commonly, potatoes) are unknown. It is known that survey data^(30)^ suggest Australians differ in their perceptions of potatoes, with roughly half of respondents agreeing that potatoes are not vegetables ‘but a carbohydrate like bread’ (consistent with the WHO definition) and the other half disagreeing or neutral (consistent with the ADG). Analysis of both the Australian and WHO definitions of vegetables allows for an understanding of vegetable choices based on choices of starchy vegetables that tend to be sweeter due to the correlation between carbohydrates and sweetness^(31)^ and human’s innate liking for sweet taste. Furthermore, such analysis can provide evidence for policy guidance in the Australian jurisdiction.

Understanding the relationship between the sensory characteristics of vegetables and consumption, how the sensory characteristics of vegetables consumed differ across age groups as a proxy for changes across age, and levels and variety of consumption may help to address the scenarios described above, inform learning strategies, guide preferred produce characteristics and identify opportunities to increase consumption. This paper seeks to address three objectives, using a large dataset from a National Nutrition Survey^(32)^ and a unique sensory database that is compatible with the national survey^(30)^. These objectives were to:

1. Understand the vegetable consumption patterns in children, with and without potatoes, using the Australian and WHO definitions.
2. Describe the sensory characteristics of vegetables consumed by children by age group, level of intake and variety.
3. Determine the vegetable preferences of children, by age group, level of intake and variety.

## Methods

Dietary intake data from the National Nutrition and Physical Activity Survey conducted as part of the 2011–12 Australian Health Survey^(32)^ was combined with an updated CSIRO Sensory-Diet database^(30)^ to examine the sensory properties of Australian children’s diets.

### National Nutrition and Physical Activity Survey data

In 2011–12, the Australian Bureau of Statistics conducted the National Nutrition and Physical Activity Survey which formed part of the Australian Health Survey. The survey reached 32 000 people from 25 000 households using a complex sampling method and design. Data were collected using a stratified multistage area sample of private dwellings. The area-based selection ensured that all sections of the population living in private dwellings within the geographic scope of the survey were represented by the sample. Furthermore, weighting these data prior to analysis means the calculated estimates better reflect the demographic structure of the Australian population and allows us to infer results for the population as a whole. Greater detail of the sampling framework is available in the comprehensive ‘Users Guide’^(33)^.

Dietary intake data were collected from 12 153 Australians (9341 adults and 2812 children). The method used to collect the dietary intake data was two, five-phase, Automated Multiple-Pass 24-h recalls, where respondents were asked to recall the previous 24-h intake of food, beverages and dietary supplements. The first recall was conducted face to face with a trained interviewer. A second dietary recall was attempted with all respondents at least 8 d later via a telephone interview. For children aged 2 years to less than 15 years, the interview was conducted primarily with a parent or guardian, and children were encouraged to participate. Parental consent was granted to interview respondents aged 15–17 years, while some parents opted to provide this information on the child’s behalf. A multiple pass approach was used which is designed to maximise respondents’ memory recall and, in conjunction with a Food Model Booklet^(34)^, assists respondents in the estimation of portion size and quantities of recalled items. For each food and beverage item recalled, nutrient intake data were derived from the matching AUSNUT database^(35)^. This database is structured into a four-tier hierarchy food classification – the most broad or ‘major’ food group (two-digit level, i.e. ‘milk products and dishes’), the ‘sub-major’ grouping (three-digit level, i.e. ‘dairy milk’), ‘minor’ grouping (five-digit level, i.e. ‘milk, cow, fluid, regular whole, full fat’) and the most descriptive ‘8-digit code’ (i.e. ‘Milk, cow, fluid, regular fat (˜3·5 %), A2’). This secondary analysis utilises data from the first 24-h recall (day 1) from 2812 children aged 2–17·9 years.

### Definition of vegetables

A range of specific definitions can facilitate an increased understanding of the influence of the sensory characteristics of diet, particularly in the case of vegetables as this broad food group is often poorly and variously defined^(36)^. This study used the WHO^(29)^ and ADG^(28)^. Both definitions included vegetable juices (only 100 % vegetables and not fruit and vegetable juice), as well as tomato products, herbs and nutritionally potent vegetables (e.g. garlic and chilli). The WHO definition excluded potatoes, sweet potatoes, cassava, yautia, taro, yams, arrowroot, sago, Jerusalem artichoke (tubers and starchy vegetables) and legumes. This also excludes potato-based mixed dishes such as potato salad but includes pumpkin, maize and green peas (sometimes described as ‘starchy’ vegetables)^(29)^. The ADG definition includes all starchy vegetables as well as legumes^(28)^. Both definitions excluded discretionary vegetables (fried starchy vegetable products) such as potato chips, deep-fried corn fritters, potato fritters, French fries or hot chips; chips or crisps. Including both definitions of vegetables allows understanding how the intake of legumes, tubers and starchy vegetables influences the sensory characteristics in a broad range of vegetables in children’s diets^(36)^.

### Identification of vegetables and vegetable-based mixed dishes containing vegetables

Vegetables and categories of vegetables were identified and re-coded at the most descriptive eight-digit level. Vegetable-based mixed dishes (three-digit code 249: mixed dishes where vegetables are the major component) were included in the analysis and included products such as vegetable stir-fry/casserole/curry and salads. However, mixed dishes where vegetables were not the major ingredient, such as vegetables in sandwiches, pasta/rice dishes, meat-based curry/stir-fry, were not included in the analysis. Therefore, a small amount of vegetables consumed within these dishes were missed, and the weight of vegetables analysed is likely to slightly underestimate total vegetable consumption. For this analysis, it was important to capture vegetables that were having an impact on the sensory characteristics of a meal or diet. Therefore, when vegetables were a minor part of a food, it was considered that sensory properties of the vegetables within these dishes would not be the dominant sensory properties of the food/meal.

Vegetables flagged as eaten in combination with other foods (i.e. combination code 9 ‘vegetables with additions’) were treated as a mixed dish. For these composite foods, a weighted sensory profile of the vegetable component was calculated as the sum of the sensory score of each component in the proportion they are present in the composite food (grams of each component/total weight of food). Discretional use of table salt was not captured as reporting could not be attributed to pairing with vegetables. Salt used in cooking (e.g. food boiled in salted water) was included when coded accordingly.

### Sensory data

The CSIRO Sensory-Diet database currently comprises over 720 foods^(37)^ and was updated to match the nutrient database (AUSNUT 2011–13) used in the 2011–13 Australian National Nutrition Survey^(30,32,35)^. Of relevance to the current study, this list of foods contained 113 vegetable and salad foods and 5 legume products and dishes. Foods were characterised by a trained sensory panel (using an adaptation of the Spectrum method)^(38)^ for five basic tastes (sweet, sour, bitter, salt and umami), as well as fatty mouthfeel, hardness, cohesiveness of mass, moistness and flavour intensity. Each modality was rated on a 100-point unstructured line scale anchored at 5 and 95 %. A systematic protocol for assigning sensory values from characterised food to other similar but un-characterised foods has been established to allow for completeness across all foods and beverages consumed by the Australian population and reflective of all foods within the nutrient composition database^(30)^. The eight-digit level code was used to join the sensory data to the corresponding food item. In taste, vegetables differ from each other mostly in the sweet and bitter dimension^(12)^ but also possess other taste qualities, for example, sour (tomato), salty (celery) and umami (mushroom).

### Statistical analysis

Statistical analyses were performed using the IBM SPSS statistical software package version 23 (SPSS Inc.). Summary estimates were weighted to reflect the demographic structure of the Australian population (based on age, sex and residential area). An additional weighting factor was applied to correct for the day of the week the survey was recorded due to disproportionately fewer recalls occurring on Sundays, and to a lesser extent Fridays. For inferential statistics, the population weights were rescaled to the size of the sample.

Vegetable consumption was examined by age group (2–3, 4–8, 9–13 and 14–18 years, age categories aligned with recommended intakes and the survey^(28,33)^), as well as by tertiles of vegetable consumption and vegetable variety (three groups: lower, medium and higher), using both Australian and WHO definitions of vegetables. Descriptive statistics included percentage of children consuming vegetables and mean vegetable intake (per day) within each group. For each sensory characteristic, the total sensory score provided by the vegetables consumed was calculated per person, and then the mean sensory score of the vegetables consumed was calculated and examined by age group, tertiles of consumption and tertiles of variety.

The differences in amount and sensory score of vegetables consumed between age groups and different levels of intake and variety (in tertiles) were tested for statistical significance using one-way between-groups ANOVA with Bonferroni *post hoc* tests. The difference in percentage consuming vegetables between age groups, consumption and variety tertile groups was tested following a chi-square test for independence. Due to the large population sample (*n* 2812), differences are highly likely to be greater than conventional measures of chance (*P* < 0·05); hence, differences are indicated when *p* < 0·01. Differences are presented below as relative and descriptive of what is reported in a large nationwide survey sample.

## Results

### Understand the vegetable consumption patterns using the Australian *v*. WHO definitions, and the contribution of potatoes to total vegetable intake

Using the definition of vegetables from the ADG (including potatoes), almost 50 % of children aged 2–3 years consumed vegetables on the day of the survey, and this decreased to 37 % in the 14–18-year-old age group (Table 1). While the percentage of children consuming vegetables decreased with age, the amount consumed (in grams) significantly increased with age (*P* < 0·001). The variety of vegetables consumed was consistent across age groups, with all children reporting about two types of vegetables on the day of the survey.

**Table 1:** Vegetable consumption (per cent consuming, mean grams consumed (sd), and mean variety score (sd)) by age group, tertiles of consumption and tertiles of variety, using the Australian Dietary Guidelines and WHO vegetable definitions

Age groups, consumption tertiles, variety tertiles	Australian Dietary Guidelines’ vegetable definition*							WHO’s vegetable definition						
	Per cent consuming (%)†	Grams of vegetables consumed		*P*-value‡	Variety of vegetables consumed		*P*-value‡	Per cent consuming (%)	Grams of vegetables consumed		*P*-value‡	Variety of vegetables consumed		*P*-value‡
		Mean	sd		Mean	sd			Mean	sd		Mean	sd	
	Broad age groups (years)							Broad age groups (years)						
2–3	49·6	108·6	96·1^a^	<0·001	1·8	1·1	0·214	41·7	90·5	81·7^a^	0·003	1·6	0·9	0·459
4–8	47·1	145·3	116·3^a^		1·9	1·2		42·2	111·5	96·9^ab^		1·7	1·1	
9–13	42·1	178·9	155·6^b^		1·9	1·2		36·1	124·5	115·4^ab^		1·6	1·0	
14–18	37·2	187·2	155·1^b^		1·8	1·2		29·6	131·5	121·4^b^		1·6	1·2	
Total	43·0	161·3	140·1		1·8	1·2		36·6	117·5	108·0		1·7	1·1	
	Tertiles of consumption							Tertiles of consumption						
Lowest		45·1	27·0^a^	<0·001	1·3	0·7^a^	<0·001		31·1	18·2^a^	<0·001	1·3	0·6^a^	<0·001
Middle		129·3	42·6^b^		1·7	0·9^b^			89·5	27·7^b^		1·6	0·9^b^	
Highest		311·4	136·3^c^		2·5	1·5^c^			235·7	112·6^c^		2·2	1·3^c^	
	Tertiles of variety							Tertiles of variety						
Lowest		119·0	122·6^a^	<0·001	1·0	0·0^a^	<0·001		90·5	93·4^a^	<0·001	1·0	0·0^a^	<0·001
Middle		163·7	118·5^b^		2·0	0·0^b^			93·3	107·0^a^		1·2	0·4^b^	
Highest		232·0	147·7^c^		3·2	1·2^c^			163·1	111·1^b^		2·7	1·1^c^	

* Australian recommendations for vegetables, legumes and beans range from 2 ½ to 5 ½ serves daily (approximately 188 g–413 g) by age and sex.

† Number of children in each age group: 2–3 years = 307, 4–8 years = 757, 9–13 years = 869, 14–18 years = 761.

‡ Significant at the *P* < 0.01 level. Superscript letters that are the same indicate no significant difference in *post hoc* analyses. Individual comparisons are shown only when overall model is significant.

Higher consumption of vegetables was associated with a greater variety of vegetables (*P* < 0·001). Children in the lowest tertile for amount consumed reported consuming 1·3 types per day, which was significantly less than 1·7 types and 2·5 types for the middle and highest tertiles of intakes, respectively (*P* < 0·001). Children in the highest tertile of vegetable variety consumed about double the amount of vegetables reported by children in the lowest tertile of variety. The percentage contribution of potatoes to total vegetable consumption increased with age. Children in the highest tertile of consumption had 30·3 % of their total vegetable intake as potatoes compared to 10·6 % for those in the lowest tertile of consumption. A similar pattern was observed by levels of variety, where the contribution of potatoes to total vegetable intake increased from 14·6 % for those with the lowest variety to 32·7 % for those with the highest vegetable variety.

Potatoes are not included in the definition of vegetables for the WHO; therefore, total vegetable consumption was lower for both percentage of children consuming and amount consumed compared to using the Australian Dietary Guideline definition (Table 1). However, the patterns of vegetable consumption described were consistent regardless of definition.

Using the WHO definition, children in the lowest tertile of intake reported 1·3 types which was less than the middle (1·6 types) and highest (2·2 types) tertiles of intakes (*P* < 0·001). The amount of vegetables consumed by children in the highest tertile of variety was slightly less than double that of children in the lowest tertile of variety. There were no statistically significantly differences in variety across age groups.

### Describe the sensory characteristics of the vegetables children consume by age group, level of intake and variety

Generally, the vegetables that children reported consuming (Table 2) were relatively bland and lacked strong tastes (below the 10th percentile of the scale), with the exception being sweetness with the strongest taste. There was little variation in the sensory profile of vegetables children reported consuming within diets by age, and no consistent linear trends of an increase or decrease in consumption with age for any of the sensory properties. The vegetables consumed within the diets of the oldest children tended to have a higher fatty mouthfeel than those in the younger age groups. The sensory properties of vegetables consumed by children in the highest tertile of consumption were higher in salty and umami tastes than those in the lowest tertile, and those in the lowest tertile higher in sour and bitter tastes. As consumption increased, the hardness of vegetables decreased and cohesiveness of mass increased. Conversely, the sensory properties of vegetables consumed by those within the lowest tertile of variety consumed vegetables that were higher in fatty mouthfeel, salty, sour and umami tastes and higher in overall flavour impact compared to those with the highest variety.

**Table 2:** Mean sensory score (sd) of vegetables consumed within the diet by age group, tertiles of consumption and tertiles of variety, using the Australian Dietary Guidelines and WHO vegetable definitions. Possible range of scores 0–100

		Hardness		Moistness		Cohesiveness of mass		Fatty mouthfeel		Sweet taste		Salty taste		Sour taste		Bitter taste		Umami taste		Overall flavour impact	
		Mean	sd	Mean	sd	Mean	sd	Mean	sd	Mean	sd	Mean	sd	Mean	sd	Mean	sd	Mean	sd	Mean	sd
Australian Dietary Guidelines	Age groups (years)																				
	2–3	29·2	15·6^ab^	44·7	11·5	31·3	8·4^ab^	6·5	6·7^ab^	19·4	8·0	6·7	7·2	4·8	6·6	5·7	4·2	3·0	5·2^ab^	32·3	6·7
	4–8	36·6	19·6^c^	45·9	10·1	31·3	8·4^a^	5·8	6·5^a^	19·7	8·8	6·9	7·6	5·9	9·4	5·8	3·7	2·2	3·8^a^	33·5	7·5
	9–13	32·9	18·6^ac^	46·5	12·0	31·1	9·5^a^	8·0	8·0^b^	18·1	8·0	8·2	9·1	6·0	8·9	5·9	3·7	3·5	5·4^b^	33·4	7·8
	14–18	27·6	16·0^b^	45·6	10·3	34·1	10·0^b^	8·7	7·6^b^	19·7	9·3	8·5	8·7	4·9	6·3	5·7	3·8	3·4	4·9^b^	32·7	7·7
	Total	32·3	18·3	45·9	11·0	31·9	9·3	7·3	7·4	19·1	8·6	7·7	8·4	5·6	8·3	5·8	3·8	3·0	4·8	33·1	7·6
	*P*-value*	<0·001		0·395		<0·001		<0·001		0·037		0·029		0·182		0·908		0·001		0·223	
	Tertiles of consumption																				
	Lowest	34·5	20·2^a^	46·5	12·2	30·6	9·7^a^	7·0	8·2	19·1	9·6	6·7	8·1^a^	6·9	10·0^a^	6·3	4·4^a^	2·5	4·4^a^	33·6	7·5
	Middle	33·4	19·7^a^	45·3	10·5	31·9	8·8^ab^	6·7	6·8	19·6	90	7·4	8·2^ab^	4·6	7·7^b^	5·4	3·6^b^	2·5	4·3^a^	32·5	8·3
	Highest	28·9	13·9^b^	45·8	9·9	33·3	9·0^b^	8·2	7·0	18·7	7·0	8·9	8·6^b^	5·1	6·4^b^	5·6	3·2^ab^	4·0	5·6^b^	33·2	6·8
	*P*-value*	<0·001		0·341		<0·001		0·011		0·342		0·001		<0·001		0·002		<0·001		0·135	
	Tertiles of variety																				
	Lowest	33·7	21·6^a^	47·2	13·3^a^	31·7	11·1	8·5	9·0^a^	19·0	10·1	9·3	10·2^a^	6·6	10·3^a^	5·6	4·3	3·9	6·0^a^	34·4	8·9^a^
	Middle	34·6	16·8^a^	44·5	7·2^ab^	31·7	7·5	5·4	4·9^b^	18·4	7·2	6·1	5·6^b^	4·6	5·6^ab^	6·1	3·2	1·8	2·5^b^	31·9	5·7^b^
	Highest	28·8	10·8^b^	44·2	7·0^b^	32·4	6·1	6·2	4·5^b^	19·7	5·9	5·5	4·3^b^	4·2	4·1^b^	6·0	3·0	2·0	2·7^b^	31·6	5·0^b^
	*P*-value*	<0·001		<0·001		0·452		<0·001		0·236		<0·001		<0·001		0·091		<0·001		<0·001	
WHO	Age groups (years)																				
	2–3	33·4	16·9^a^	49·3	9·4	27·8	6·6	4·4	6·3^ab^	22·6	8·9	5·2	6·8	5·5	8·3	5·9	4·5	1·8	3·8	34·6	7·3
	4–8	40·3	18·8^b^	48·7	9·5	27·6	6·1	3·7	5·6^a^	21·9	9·3	6·0	7·2	6·3	9·7	6·3	4·4	1·5	3·1	35·4	6·7
	9–13	37·0	18·7^ab^	49·4	11·9	27·4	8·0	5·4	7·4^b^	20·6	8·8	6·0	7·9	6·7	9·3	6·8	4·5	2·3	4·4	34·9	6·9
	14–18	32·2	17·1^a^	49·3	10·4	28·2	6·6	5·5	7·2^ab^	22·2	9·2	5·7	6·9	5·3	7·4	6·2	4·3	2·1	4·0	34·0	7·5
	Total	36·5	18·4	49·2	10·5	27·7	6·9	4·7	6·7	21·6	9·1	5·8	7·3	6·1	9·0	6·4	4·4	1·9	3·8	34·8	7·1
	*P*-value*	<0·001		0·837		0·594		0·003		0·082		0·727		0·258		0·194		0·071		0·181	
	Tertiles of consumption																				
	Lowest	36·6	18·7	48·4	11·4	27·8	6·3	5·0	7·6	19·3	9·6^a^	5·1	6·1^a^	6·5	9·8	7·4	5·2^a^	1·6	3·4^a^	33·5	7·2^a^
	Middle	35·3	18·5	49·8	9·7	27·9	6·6	4·3	5·8	22·4	8·8^b^	5·1	6·0^a^	6·3	9·7	5·9	4·1^b^	1·6	2·9^a^	34·3	6·9^a^
	Highest	37·7	18·0)	49·3	10·3	27·4	7·8	4·9	6·6	23·1	8·4^b^	7·4	9·2^b^	5·5	7·0	5·8	3·8^b^	2·7	4·9^b^	36·7	6·7^b^
	*P*-value*	0·256		0·219		0·683		0·377		<0·001		<0·001		0·331		<0·001		<0·001		<0·001	
	Tertiles of variety																				
	Lowest	38·2	20·6	50·3	12·5^a^	27·6	8·0	5·0	7·6	20·7	10·3^a^	6·6	8·6^a^	7·0	11·1^a^	6·3	4·8	2·1	4·2	35·0	8·2
	Middle	33·9	20·7	49·7	10·3^ab^	27·8	6·2	5·6	7·6	21·4	8·9^ab^	5·7	7·8^ab^	5·6	8·8^ab^	5·7	4·4	2·1	4·5	33·7	8·1
	Highest	35·4	13·5	47·4	6·8^b^	27·8	5·5	4·1	4·8	22·9	7·2^b^	4·9	4·8^b^	5·1	4·7^b^	6·7	3·8	1·7	2·9	35·0	4·4
	*P*-value*	0·016		<0·001		0·848		0·032		0·003		0·004		0·008		0·057		0·184		0·106	

* Significant at the *P* < 0.01 level. Superscript letters that are the same indicate no significant difference in *post hoc* analyses. Individual comparisons are shown only when overall model is significant.

The fatty and sweetness of the vegetables reported was lower using the Australian definition than the WHO definition (when potatoes are excluded) at all levels of consumption. Similar findings for hardness and fatty mouthfeel by age group were found, in that hardness decreased from ages 4–8 to 14–18 years age groups; and fatty mouthfeel was higher in the oldest two age groups. Interestingly when potatoes are excluded, sweet taste was significantly higher and overall flavour impact significantly higher in the highest consumers compared to the lowest consumers.

### Determine the vegetable preferences of children, by age group, level of intake and variety

To understand the types of vegetables contributing to the differences in sensory properties of vegetables in the diet by age group, we examined the most commonly consumed vegetables and their contribution to total vegetable intake (Tables 3 and 4). When using the Australian definition (Table 3), across all age groups, potatoes were the most commonly consumed vegetable, accounting for 14·9–21·2 % of total consumption, depending on age. Potatoes were followed by carrots (most commonly, cooked) in all age groups except 2–3-year olds (overall 11·6 % of total vegetables). Other commonly consumed vegetables included sweetcorn (6·3 % of total consumption overall), broccoli and cauliflower (6·4 %), and mixtures of vegetables (8·2 %). Potato-mixed dishes (e.g. potato bake), legumes and dishes with legumes (e.g. baked beans) were common among both the lowest tertile of variety and also the highest tertile, suggesting variety is modest at all levels and consumption is dominated by a few types regardless of intakes, that is, among the top five most commonly consumed vegetables, independent of age group.

**Table 3:** Top five ranked vegetables based on number of serves consumed (per cent of total intake), by age group, level of vegetable intake and variety of vegetables consumed using the Australian dietary guidelines definition of vegetables†

		Potatoes		Carrots		Mixed vegetables		Sweetcorn		Broccoli, broccolini and cauliflower		Other fruiting vegetables		Legume-based dishes		Potato mixed dishes		Legume and pulse products		Vegetable based salads		Vegetables and sauce	Tomato		Peas/podded peas
**Australian Dietary Guidelines**	**Age group (years)**																								
	2–3	1	14·9 %	4	10·2 %	3	10·4 %	5	9·5 %					2	11·8 %										
	4–8	1	20·6 %	2	14·4 %	4	8·7 %	3	9·5 %	5	8·3 %														
	9–13	1	20·4 %	2	10·4 %	3	7·2 %							4	7·2 %	5	6·1 %								
	14–18	1	21·2 %	2	10·8 %	4	8·2 %									5	7·0 %	3	9·4 %						
	Total	1	20·2 %	2	11·6 %	3	8·2 %	5	6·3 %	4	6·4 %														
	**Tertiles of consumption**																								
	LOWEST	4	7·8 %	1	19·3 %	2	12·7 %													3	12·2 %		5	7·8 %	
	MIDDLE	1	21·5 %	3	12·3 %	2	12·6 %	4	8·7 %											5	8·1 %				
	HIGHEST	1	21·5 %	2	10·1 %					3	7·2 %			4	6·3 %	5	6·1 %								
	**Tertiles of variety**																								
	LOWEST	1	12·0 %	5	8·5 %	2	11·1 %							3	9·9 %			4	9·8 %						
	MIDDLE	1	21·5 %	3	11·4 %	2	13·1 %			4	11·2 %	5	7·7 %												
	HIGHEST	1	26·6 %	2	14·3 %			4	7·6 %	3	8·8 %					5	5·2 %								

† Serves were estimated using 75g/serve.

**Table 4:** Top five ranked vegetables based on number of serves* consumed (per cent of total intake), by age group, level of vegetable intake and variety of vegetables consumed using the WHO definition of vegetables

		Potatoes	Carrots		Mixtures of two or more vegetables		Sweetcorn		Broccoli, broccolini and cauliflower		Other fruiting vegetables		Dishes where mature legumes are the major component	Potato mixed dishes	Legume and pulse products	Salads, vegetable based		Vegetables and sauce		Tomato		Peas and edible-podded peas	
			*n*	%	*n*	%	*n*	%	*n*	%	*n*	%				*n*	%	*n*	%	*n*	%	*n*	%
**WHO**	**Age group (years)**																						
	2–3	n/a	2	16·3 %	1	16·7 %	3	15·3 %	5	9·7 %	4	10·7 %	n/a	n/a	n/a								
	4–8		1	21·1 %	3	12·8 %	2	13·9 %	4	12·2 %	5	10·5 %											
	9–13		1	17 %	2	11·8 %			4	8·6 %	5	8·5 %						3	8·8 %				
	14–18		1	19·6 %	2	14·9 %	5	8·6 %	3	11·1 %						4	9·2 %						
	Total		1	18·9 %	2	13·4 %	4	10·2 %	3	10·4 %	5	7·7 %											
	**Tertiles of consumption**																						
	LOWEST		3	13·3 %	2	19 %			4	6·5 %				1	20·7 %							5	6·5 %
	MIDDLE		1	22 %	2	18·4 %			4	8·2 %				3	11·3 %					5	6·9 %		
	HIGHEST		1	18·3 %	4	10·4 %	2	13 %	3	12 %	5	8·8 %											
	**Tertiles of variety**																						
	LOWEST		2	14·7 %	1	22 %	5	8·6 %						3	10·6 %	4	8·8 %						
	MIDDLE		2	14·9 %	1	21·1 %	4	7·8 %	5	6·8 %				3	12·1 %								
	HIGHEST		1	22·5 %	5	6 %	3	11·9 %	2	15·6 %	4	8·3 %											

* Serves were estimated using 75g/serve.

For children in the lowest tertile of consumption, carrots were the most commonly consumed vegetable, followed by vegetable mixtures, salad and then potatoes (ranked 4). For children in the highest tertile of consumption, the most commonly consumed vegetables were potatoes, followed by carrots, broccoli and cauliflower, legume dishes and potato-mixed dishes. In the highest vegetable consumers, 27·6 % of total vegetable intake was from potatoes or potato-based dishes. Regardless of variety level, potatoes were the most commonly consumed vegetable.

Using the WHO definition (Table 4), cooked carrots were the most commonly consumed vegetable for those with highest consumption and with highest variety. Salads were displaced by cooked vegetables for lower consumers. The vegetables appearing in the five most frequently consumed was largely consistent by age group, and these top five vegetables accounted for almost two-thirds of consumption (˜60 %) among the lowest and highest tertiles of consumption, suggesting variety is modest at all levels and consumption is dominated by a few types regardless of intakes.

## Discussion

The aims of this paper were to understand the sensory characteristics of vegetables consumed (with and without the inclusion of potatoes) by Australian children and to determine whether vegetable preferences of children varied by the amount or variety of vegetables consumed. The implications of the results for each aim are discussed in more detail.

### Vegetable consumption patterns using the Australian v. WHO definitions, and the contribution of potatoes to total vegetable intake

The current study was a secondary analysis of a nationally representative sample of Australian children to explore the sensory properties of vegetables consumed and how this varies by age group, and by the amount and variety consumed, to reveal sensory-focused opportunities to increase consumption. Children’s vegetable consumption increased with age, but variety was constant, suggesting that older children are achieving higher consumption by eating larger portions of a narrow range of vegetables, as opposed to more variety.

Australian children reported to consume a narrow range of vegetables. Potatoes were the most commonly consumed, and the top five vegetables contributed to two-thirds of total vegetable intake. With increased total vegetable consumption, the contribution of potatoes increased. The inclusion of potatoes (and other starchy roots and tubers) in the ADG is in alignment with the choice children reported. This choice of potatoes is possibly related to their energy content^(39)^, ease of eating (high cohesiveness of mass) and relatively bland sensory qualities (low taste levels and low overall flavour impact) and low cost. There would seem to be a ‘natural’ tendency for children (or their parents/carers) to choose potatoes, suggesting that the inclusion of potatoes within the ADG may require careful consideration relative to the encouragement of variety, which is also part of recommendations of the ADG and most other jurisdictions around the world, which promote the importance of variety. Given that knowledge of dietary guidelines is poor^(40)^, greater emphasis on the importance of consuming a variety of vegetables for the beneficial health effects as well as a means to achieving greater overall intake is needed. Low variety may be related to limited variety presented in infancy. There is some evidence that exposure to variety is particularly important among infants^(41)^ and young children^(42)^ in encouraging overall intakes (in contrast to specific targeted vegetables). A recent study on aspects of the same Australian dataset as the current study found that most Australian young adults also failed to consume a variety of (fruits and) vegetables, with those in the youngest age group (18–24 years), contiguous with the current dataset’s oldest age group, reporting the lowest intakes and variety^(43)^. This suggests that the lack of variety and associated low consumption extends into adulthood.

### The sensory characteristics of the vegetables children consume by age group, level of intake and variety

Commonly consumed vegetables were relatively bland and lacking in strong tastes, with sweetness the dominant taste. Comparing the values in Table 2 of the current paper (using WHO definition of vegetables) with Table 2 of Poelman^(12)^ provides evidence that children consume relatively sweet and less bitter vegetables compared to the range of vegetables available.

When potatoes were included (increasing proportionally as consumption increased), the hardness of vegetables generally decreased and cohesiveness of mass increased, with these properties tending to positively favour ease of eating (lower hardness requires less chewing and greater cohesiveness forms a bolus more easily, so less time is required to prepare for a safe swallow). These findings can be explained by the higher intake of potatoes in the older age groups, which is supported by a lack of difference in these properties with age when using the WHO definition of vegetables. Counter-intuitively, younger age groups’ vegetable choices for higher ‘hardness’ suggests that choices are not influenced by deciduous dentition and confirm small-scale studies suggesting young children’s preferences for ‘crunchiness’ (hardness)^(44,45)^.

The absence of differences in the sensory characteristics of vegetables across age groups is consistent with the effects of early exposure sustained throughout life^(26)^. Children follow innate preferences for sweeter tasting vegetables (with or without the inclusion of potatoes), and these innate preferences are associated also with higher intakes (essentially, more of the same sensory qualities). Our data provide additional evidence in support of a recent study suggesting preferences for ‘more appealing’ sensory characteristics among European adolescents^(8)^. In that study, secondary data were used to categorise selected vegetables into dichotomised ‘more’ or ‘less’ appealing sensory characteristics by the researchers; however, those assumed categorisations are broadly in line with the more robust data presented in the current study, for example, sweet and bland (delicate flavour). Nevertheless, capitalising on innate sensory preferences to encourage consumption may be a short-term strategy worth pursuing, albeit with limited effect on total intakes.

High consumers consumed more salty (and umami) vegetables. This finding may have been due to the consumption of vegetable-based mixed dishes which tend to include salt-containing sauces. Currently, there is limited evidence for the benefits of addition of salt on vegetable intake. One study^(45)^ has investigated the effect of flavour additions on acceptance of green beans in 2–3-year olds. Added salt (0·6 %) increased intake of green beans, whereas added fat (2·5–5 %) did not. Salt (0·6–1·2 %) increased salty taste but did not decrease bitter taste significantly despite other evidence suggesting that salt can suppress the perception of bitterness generally^(46,47)^. There is emerging evidence that children are more sensitive to bitter taste than adults^(15)^, but it is unknown as to whether salty taste might encourage the acceptance of bitter-tasting vegetables among children. The data presented in the current study suggest that salty taste may play a positive role and the relative benefits of increased vegetable intakes could outweigh the health risks of additional salt. Low-intake consumers consumed more sour vegetables than high-intake consumers. This may potentially be explained by higher consumption of tomatoes, since salads and mixed vegetable dishes were amongst the most frequently consumed vegetable categories for this group and not for the high-intake group. They also consumed less bitter vegetables, which may be related to perceptual suppression of salty taste on bitter taste^(46,47)^.

### Scenarios explored: learning effects

The introduction to this study provided scenarios that were explored, including an exploration of learning effects with age. The data do not provide evidence that as children age, they learn to like a variety of vegetables with diverse and innately disliked sensory characteristics, for example, bitter taste. Taking the cross-sectional age cohort data as a proxy for ageing, there is no evidence that younger children eat more vegetables that align with innate likings, that is, sweeter vegetables and that this changes with age, exposure and consumption level. Indeed, it does not apply to (small) increases in variety either. Hence there is no evidence of a learning effect. This suggests that more effort and support is needed to assist with learning through exposure to a variety of vegetables, particularly at younger ages. The evidence for effectiveness of exposure strategies is emerging^(24)^, but studies often focus on a few select vegetables or one target vegetable. Exposure to a narrow range does not generalise to acceptance of the whole diverse category of vegetables. Furthermore, studies suffer from (the challenging) lack of outcome effects on usual vegetable intakes and often focus upon short-term effects of acceptance of target vegetables. There is a need for strategies and studies that include exposure, variety and effects on usual vegetable intakes.

### Limitations

Dietary intake data for the Australian National Nutrition Survey were collected through 24-h recall. While considered a robust measure of intake, all dietary assessment methods are impacted by misreporting and social desirability to some degree. Social desirability would suggest healthy foods like vegetables would more likely be over-reported relative to true consumption, but the low intakes reported suggest that any biases are minimal. Taking the cross-sectional age cohort data as a proxy for ageing is a further limitation. There is an absence of longitudinal data. Usage of age cohorts is common as a proxy for age effects. Furthermore, the stability of low vegetable intakes among Australian children in recent years^(5)^ suggests a lack of cohort effects on intakes. Nevertheless, care should be taken in interpreting the robustness of the lack of learning effects with age. Longitudinal studies are needed to confirm or disprove these findings. For this study, vegetables were identified by discrete food item codes, including when vegetables were the largest component of mixed dishes, and the sensory properties of the identified foods analysed. We cannot say how other foods and condiments consumed at the same occasion as vegetables influenced perception of foods eaten together. Nevertheless, a strength of the current study was that detailed information on the sensory characteristics of vegetables children reported eating were objectively measured. The sensory database was developed using a trained adult panel as an ‘objective’ (calibrated) measure of the sensory qualities of foods. This is a gold standard used by sensory science and the food industry, but it does not reflect the possible differences in perception by children. Children’s limited abilities to rate sensory qualities of foods are well documented^(48,49)^.

## Conclusions

Australian children’s vegetable consumption is below recommendations and variety is lacking. If cross-sectional age cohorts are a proxy for what happens as children age, then changes in consumption patterns over time are modest and support the theory that early exposure tends to determine what happens in later life. Increased consumption of vegetables with age was largely driven by higher potato consumption. When potatoes are included in the definition of vegetables, their influence is marked across many sensory modalities imposing further moderating effects on a bland food category. When potatoes were not included, fatty mouthfeel, salty, sweetness and umami increased with increasing vegetable consumption. Not surprisingly variety has the most marked effect on sensory differences, although, by both definitions, children’s vegetable variety was modest, with the top five vegetables accounting for nearly two-thirds of total intake. These data suggest that there should be considerable encouragement of consuming a variety of vegetables at earlier ages and sustained across all ages, as variety was associated with greater total vegetable consumption.
